# Computer-aided dermoscopy for diagnosis of melanoma

**DOI:** 10.1186/1471-5945-5-8

**Published:** 2005-07-06

**Authors:** Masoomeh Barzegari, Haiedeh Ghaninezhad, Parisa Mansoori, Arash Taheri, Zahra S Naraghi, Masood Asgari

**Affiliations:** 1 Department of Dermatology, Razi Hospital, Tehran University of Medical Sciences, Vahdat-Eslami Street, 11996 Tehran, Iran; 2 Department of Pathology, Razi Hospital, Tehran University of Medical Sciences, Vahdat-Eslami Street, 11996 Tehran, Iran

## Abstract

**Background:**

Computer-aided dermoscopy using artificial neural networks has been reported to be an accurate tool for the evaluation of pigmented skin lesions. We set out to determine the sensitivity and specificity of a computer-aided dermoscopy system for diagnosis of melanoma in Iranian patients.

**Methods:**

We studied 122 pigmented skin lesions which were referred for diagnostic evaluation or cosmetic reasons. Each lesion was examined by two clinicians with naked eyes and all of their clinical diagnostic considerations were recorded. The lesions were analyzed using a microDERM^® ^dermoscopy unit. The output value of the software for each lesion was a score between 0 and 10. All of the lesions were excised and examined histologically.

**Results:**

Histopathological examination revealed melanoma in six lesions. Considering only the most likely clinical diagnosis, sensitivity and specificity of clinical examination for diagnosis of melanoma were 83% and 96%, respectively. Considering all clinical diagnostic considerations, the sensitivity and specificity were 100% and 89%. Choosing a cut-off point of 7.88 for dermoscopy score, the sensitivity and specificity of the score for diagnosis of melanoma were 83% and 96%, respectively. Setting the cut-off point at 7.34, the sensitivity and specificity were 100% and 90%.

**Conclusion:**

The diagnostic accuracy of the dermoscopy system was at the level of clinical examination by dermatologists with naked eyes. This system may represent a useful tool for screening of melanoma, particularly at centers not experienced in the field of pigmented skin lesions.

## Background

The incidence of melanoma is much lower in Asia than in western countries [1]. Clinicians sometimes misdiagnose early melanoma especially in areas with lower incidence of disease [2]. Because advanced cutaneous melanoma is still incurable, early detection by means of accurate screening is an important step towards a reduction in mortality.

Recently, computer-aided dermoscopy using artificial neural networks (ANNs) has been reported to be an accurate tool for the evaluation of pigmented skin lesions (PSLs) [3-5].

To our knowledge, the accuracy of such system for diagnosis of PSL has not been demonstrated in the Middle East, where most of the patients have Fitzpatrick skin type III-IV. We set out to determine the sensitivity and specificity of this system in Iranian patients.

## Methods

One hundred and twenty two consecutive PSLs equal or smaller than 15 mm in diameter, with a clinical diagnosis of one of the pigmented melanocytic lesions, which were referred to dermatology clinic of Razi Hospital for diagnostic evaluation or cosmetic reasons were included in the study. Each lesion was examined by two clinicians (an attending dermatologist and a third year dermatology resident) with naked eyes. They consulted with each other and recorded all of their clinical diagnostic considerations in a list. The first diagnostic consideration in the list was the most likely clinical diagnosis. After clinical examination, the lesions were analyzed using a microDERM^® ^dermoscopy unit. The system consists of a special camera, which had ability to take images at ×15, ×20, ×30, and ×50 magnifications and contains a 752 × 582 pixel charge-coupled device. The image analysis software was Visiomed AG (Ver. 3.50) based on an ANN that was trained using images collected in a Europe-wide multicenter study (DANAOS) [6]. The output value of the software is a score ranging from 0 to 10 for each lesion. Informed consent was obtained from each patient. All of the lesions were excised and examined histologically. The final diagnosis was made based on pathological examination.

The study was approved by the Research Ethics Committee of Razi Hospital.

## Results

One hundred and twenty two pigmented skin lesions from 91 Iranian patients (30 male, 61 female; mean age 32.3 years, age range 6–94) were included in the study.

Melanoma was in the list of clinical diagnostic considerations in 19 lesions. It was the most likely clinical diagnosis in nine lesions.

Table 1 shows the frequency and dermoscopy score of the PSLs. Histopathological examination revealed melanoma in six lesions.

**Table 1:** Frequency and dermoscopy score of the lesions according to histopathological diagnosis.

Histopathological diagnosis	No. of lesions		Dermoscopy Score	
	
	N	%	Mean	Range
**Melanoma**	**6**	**4.9**	**8.05**	**(7.34–8.47)**
Lentigo Maligna	3	2.5	7.90	(7.34–8.47)
Lentigo maligna melanoma	2	1.6	8.19	(7.92–8.47)
Acral lentiginous melanoma	1	0.8	8.24	-----
**Nonmelanoma**	**116**	**95.1**	**3.86**	**(0.00–8.35)**
Melanocytic	111	91	3.74	(0.00–8.35)
Junctional nevus	4	3.2	2.54	(1.92–3.21)
Compound nevus	10	8.1	2.86	(1.84–5.23)
Intradermal nevus	76	62.3	3.68	(0.00–8.35)
Congenital nevus	7	5.7	5.34	(0.54–7.52)
Blue nevus	5	4.1	2.24	(0.35–3.72)
Combined nevus	2	1.6	4.54	(1.39–7.69)
Dysplastic nevus	7	5.7	5.66	(1.70–8.06)
Nonmelanocytic	5	4.1	6.42	(0.64–8.09)
Seborrheic keratosis	2	1.6	8.00	(7.91–8.09)
Dermatofibroma	1	0.8	7.96	-----
Epidermal nevus	1	0.8	0.64	-----
Actinic keratosis	1	0.8	7.53	-----
**Total**	**122**	**100**	**4.06**	**(0.00–8.47)**

N, number

Considering only the most likely clinical diagnosis, sensitivity and specificity of clinical examination for diagnosis of melanoma were 83% and 96%, respectively. Considering all clinical diagnostic considerations, the sensitivity and specificity were 100% and 89%.

**Figure 1:**
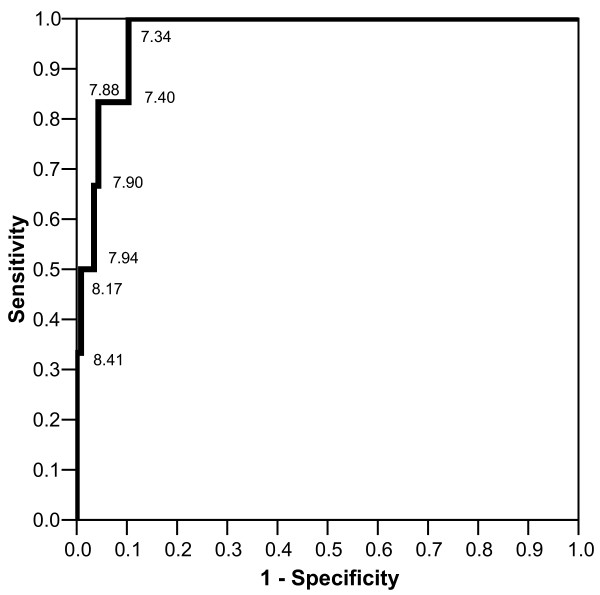
Receiver operating characteristic curve for the separation of benign pigmented skin lesions and melanoma using dermoscopy score.

Figure 1 shows receiver operating characteristic (ROC) curve for the separation of benign PSLs and melanoma using dermoscopy score in our study. In order to compare the sensitivity and specificity of clinical examination with dermoscopy score, we selected two points on the ROC curve that showed sensitivity and specificity near that of clinical examination. Choosing a cut-off point of 7.88 for dermoscopy score, the sensitivity and specificity of the score for diagnosis of melanoma were 83% and 96%, respectively. Setting the cut-off point at 7.34, the sensitivity and specificity were 100% and 90%.

Cohen's kappa statistic was used for evaluation of agreement between diagnostic tests. Kappa values are shown in Table 2.

**Table 2:** Cohen's kappa values for evaluation of agreement between diagnostic tests.

Tests	Kappa value
PD versus CD1	0.65
PD versus CD2	0.44
PD versus DS1	0.50
PD versus DS2	0.46
CD1 versus DS1	0.64
CD2 versus DS2	0.65

CD1, clinical diagnosis when considering only the most likely clinical diagnosis; CD2, clinical diagnosis when considering all clinical diagnostic considerations; DS1, dermoscopy score when setting the cut-off point at 7.88; DS2, dermoscopy score when setting the cut-off point at 7.34; PD, pathological diagnosis.

## Discussion

The ABCD rule is one of the most widely used methods for evaluating PSLs with the naked eye. However, the diagnostic accuracy is not very high [7,8].

Dermoscopy is a noninvasive method that enables clinicians to evaluate numerous morphological features that are not visible to the naked eye. Several studies have shown that this method improves diagnostic accuracy by 20–30% compared with simple clinical observation [9-11]. Recently, computer-aided dermoscopy has been introduced as an additional tool to improve the diagnosis of pigmented skin lesions. In previous reports, the sensitivity of digital dermoscopy analysis for diagnosis of melanoma has ranged between 80% and 100% [12,13]. The specificity has ranged between 46% and 98% [14,15]. The diagnostic accuracy of most of the reported softwares have been at the level of a dermatologist experienced in dermoscopy and higher than inexperienced clinicians [3-5,16]. In one study carried out by Seidenari et al, diagnostic accuracy of the software was higher than clinical assessment by an experienced observer [17]. They reported that the sensitivity and specificity of computer analysis for diagnosis of melanoma were 93% and 95%, respectively. In that study, clinical assessments were performed on 20-fold magnified images. Sensitivity and specificity of clinical diagnosis made by an experienced observer were 81% and 95%. When an untrained dermatologist assessed the images, sensitivity and specificity of clinical diagnosis were 74% and 75%. Another study carried out by Bauer et al revealed that diagnostic accuracy of computer-aided dermoscopy was higher than dermoscopy by a trained dermatologist [15].

Visiomed AG, our image analysis software, has been trained using images collected from European countries. However, it could detect melanoma in Iranian patients with a high level of accuracy. The accuracy of the computerized dermoscopy system in our study is comparable with that of the most accurate reported systems [5,6,16].

Comparing clinical examination with dermoscopy score for diagnosis of melanoma in our study, there is no considerable difference in sensitivity and specificity. However, a larger study with higher power may detect a possible difference.

## Conclusion

This system could not help us to reduce unnecessary excisions or improve early melanoma detection. Nevertheless, it may improve the diagnostic accuracy of an inexperienced clinician in the clinical evaluation of PSLs and represent a useful tool for screening of melanoma, particularly at centers not experienced in the field of PSLs. However, the cost benefit ratio of using this system needs to be assessed in developing countries with a low incidence of melanoma. Furthermore, it is of paramount importance to clarify that computer analysis has been developed in order to assist and not to replace physicians in the diagnosis of PSLs.

## Competing interests

The author(s) declare that they have no competing interests.

## Authors' contributions

MB participated in the design of the study and oversaw the drafting process and gave critical inputs to the manuscript.

HG participated in the design of the study, evaluation of the patients, and dermoscopic analysis of the lesions and oversaw the drafting process and gave critical inputs to the manuscript.

PM participated in the design of the study, evaluation of the patients, dermoscopic analysis of the lesions, histological evaluation of the lesions, statistical analysis, and drafting the manuscript.

AT participated in the design of the study, statistical analysis, and drafting the manuscript.

ZSN participated in histological evaluation of the lesions.

MA participated in histological evaluation of the lesions.

All authors read and approved the final manuscript.

## Pre-publication history

The pre-publication history for this paper can be accessed here:


